# Spatio-spectral localized modal coupling for room-temperature quantum coherence protection

**DOI:** 10.1515/nanoph-2024-0574

**Published:** 2025-03-20

**Authors:** Wen-Jie Zhou, Yu-Wei Lu, Jing-Feng Liu, Renming Liu, Lay Kee Ang, Ortwin Hess, Lin Wu

**Affiliations:** Department of Science, Mathematics and Technology (SMT), 233793 Singapore University of Technology and Design (SUTD), 8 Somapah Road, Singapore 487372, Singapore; Department of Electrical and Computer Engineering, National University of Singapore, 4 Engineering Drive 3, Singapore 117583, Singapore; Quantum Science Center of Guangdong–Hong Kong–Macao Greater Bay Area (Guangdong), 518045, Shenzhen, China; College of Electronic Engineering and College of Artificial Intelligence, South China Agricultural University, 510642, Guangzhou, China; School of Physics and Electronics, International Joint Research Laboratory of New Energy Materials and Devices of Henan Province, Henan University, 475004, Kaifeng, China; School of Physics and CRANN Institute, Trinity College Dublin, Dublin 2, Ireland

**Keywords:** modal coupling, spatio-spectral localized, surface lattice resonance, localized surface plasmonic resonance, spectral hole-burning, coherence

## Abstract

This work aims to advance the room-temperature manipulation of photonic qubits and enhance coherence preservation in and for quantum applications via tailored spatio-spectral localized (SSL) systems. We focus on an innovative all-plasmonic SSL system consisting of a gold bowtie array on a gold substrate. This design produces a high-Q spectral-localized mode through the lattice array, emerging from the collective lattice response of localized surface plasmon resonance (LSPR), particularly the surface lattice resonance (SLR). The SSL system enables tunable modal coupling between the LSPR and SLR, allowing precise alignment with quantum emitters to form quasi-bound states across an energy range of 1.45–1.91 eV. This flexibility allows us to investigate how innovative configurations – such as three-body coupling symmetry and modal-coupling strength – affect coherence protection. These insights pave the way for optimizing SSL systems, setting the stage for significant advancements in nanophotonic qubit manipulation at ambient conditions and the future of photonic quantum systems.

## 1 Introduction

Coherence time is a critical parameter in quantum technologies, influencing the precision and effectiveness of qubit manipulation [1], [2]. Traditional quantum computing systems utilizing dielectric photonic cavity designs [3], [4], [5], [6] following principles of cavity quantum electrodynamics (cQED) typically operate at cryogenic temperatures to achieve sufficient coupling strength for effectively “capturing” qubits, a process that can be both energetically costly and inefficient. In contrast, plasmonic nanoantennas and nanocavities facilitate quantum manipulation at ambient conditions [7], [8], [9], [10], [11], leveraging their resonant modes with significantly reduced mode volumes and enhanced intensities. These concentrated mode fields create “hotspots” where a high local-density-of-states (LDOS) can effectively “capture” quantum emitters (QEs) and promote the formation of plexcitons for manipulation [11], [12]. However, the benefits of nanoplasmonic spatial localization are partly offset by intrinsic metallic losses, which can lead to decoherence, primarily due to dissipation [13], [14], [15].

Recently, the concept of quantum nanoplasmonic coherent perfect absorption (qnCPA) [16] has been introduced. Leveraging an inherent frequency specificity to selectively initialize a coupled system in a chosen plasmon-emitter dressed state, it has been demonstrated that qnCPA allows (through a coherent, unidirectional, and non-perturbing near-field energy transfer from a proximal plasmonic waveguide) to render a plexciton state robust against dynamic dissipation under ambient conditions [16].

An alternative strategy for enhancing coherence in cQED systems is to hybridize plasmonic nanoantennas with dielectric microcavities, creating “optoplasmonic” systems [14], [17]. Examples include plasmonic nanoparticles hybridized with polystyrene beads [18], plasmonic nanorods coupled with whispering-gallery-mode microcavities [19], and long-range surface plasmons interacting with waveguide modes [20]. These systems utilize high-quality (high-Q) photonic modes for spectral localization, acting as capacitors to protect coherence [20], [21], [22], [23]. Notably, atom-photon quasi-bound states (qBS) in plasmonic-dielectric hybrids [24] can suppress decoherence through unique eigenstates that exhibit low decay rates, similar to the Friedrich–Wintgen quasi-bound states in the continuum (quasi-BIC) found in photonic systems [25]. This suppression of decoherence is crucial for enhancing quantum system performance by prolonging coherence times. However, implementing these hybrid designs presents significant experimental challenges, particularly in achieving qBS under specific coupling conditions. For instance, in structures like gold nanorod dimers on nanobeams [26], precise alignment is essential: the dimer must be accurately centered within the photonic taper, and the QE must be positioned exactly at the center of the dimer. This meticulous positioning requires advanced techniques [27], [28]. One potential solution is integrating all components on a chip [29], [30], though this approach still faces challenges in achieving precise overlay accuracy across multiple materials.

These challenges can be effectively addressed through plasmonic lattice designs [31], [32], [33], which enable one-stop fabrication in a single material platform. This approach eliminates the need for precise repositioning or complex integration, allowing QEs to be spin-coated onto the surface for collective signal measurement. Importantly, lattice arrays can support high-Q surface lattice resonance (SLR) modes (>2000) for spectral localization [34], [35], stemming from the collective resonances of the lattice nanostructures. While several plasmonic lattice designs have shown strong modal coupling between SLR and localized surface plasmon resonances (LSPR) [32], [36], [37], typically using single nanoparticle arrays, they often lack optimal spatial localization of LSPR. A notable optical metasurface achieved strong modal coupling between plasmonic gap modes and photonic lattice modes in DNA-assembled gold nanocube arrays. This approach combines top-down and bottom-up processes, where gold nanocubes are organized into arrays via DNA hybridization on a gold film patterned with DNA-binding regions defined by electron beam lithography [37].

Here, we introduce the concept of a spatio-spectral localized (SSL) system, where modes are considered localized only if their spatial or spectral confinement matches the size or bandwidth of the QE. This concept highlights the system’s potential for quantum manipulation under ambient conditions and demonstrates the generation of a quasi-bound state (qBS) through QE-SSL hybridization. In particular, we present and discuss an experimentally accessible plasmonic metasurface platform that achieves SSL via LSPR-SLR modal coupling, allowing easy tailoring to align with specific QEs. To analyze this SSL system quantitatively, we develop a comprehensive modal-coupling model that extracts modal-coupling parameters from electric field measurements. These modal parameters are applied in a generalized three-body quantum model, where a practical QE, *e*.*g*., excitons in monolayer MoS_2_, interacts with the SSL system. The resulting spectral intensity and dynamics illustrate how LSPR spatially captures the QE while SLR spectrally stores quantum coherence, effectively forming a qBS. Beyond the exemplified LSPR-SLR modal coupling, we explore coupling symmetry breaking and variations in coupling strength to provide general insights into QE-SSL hybridization and quantum coherence protection. It should be noted that this work focuses exclusively on dissipation while neglecting other decoherence effects such as dephasing and depolarization. Overall, our goal is to generalize the concept of SSL modal coupling for broader applications in plasmonic cQED systems.

## 2 Theory and concept

**Figure 1:**
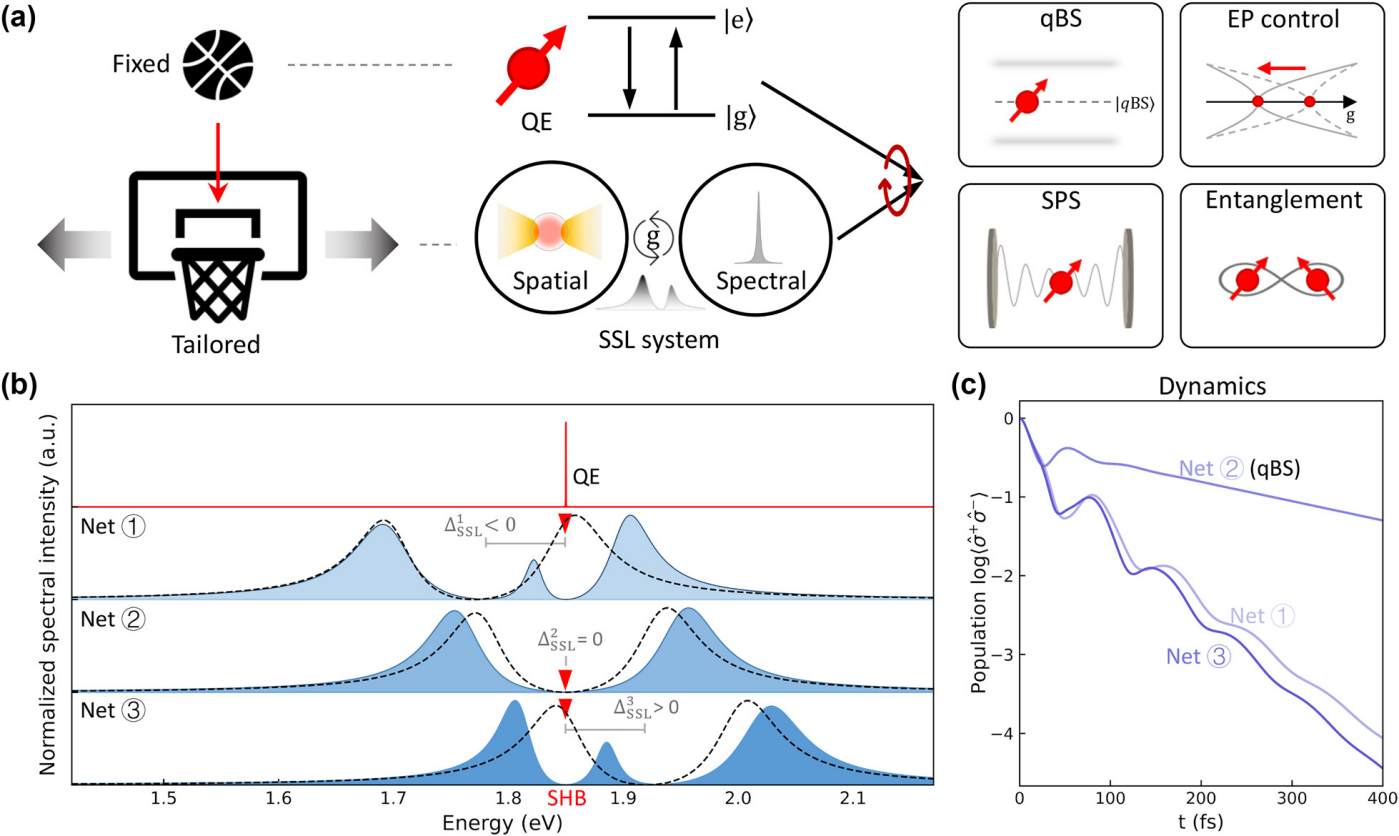
Spatio-spectral localized (SSL) modal coupling for atom-photon quasi-bound states (qBS) (a) schematic of the matching process involving a fixed quantum emitter (QE) modeled as a two-level system and an SSL system with a broad spatial-localized mode and a narrow spectral-localized mode, enabling various quantum applications. (b) Three different SSL systems with strong modal coupling (dashed lines), exhibiting spectral splitting, are tuned around a QE oscillating at *ω*_e_ = 1.85 eV, represented by tailored nets 1, 2, and 3. The QE (red triangles) induces spectral hole-burning (SHB) in the SSL-QE hybrid systems (blue-shaded). (c) Dynamics of the QE in the three tailored SSL systems in (b). The Net 2, identified as a qBS, allows the longest coherence lifetime of the QE.

To score in an imaginary game of basketball, one strategy would be to align the ball with a stationary net, while an alternative approach would involve moving the net to accommodate the (oncoming) ball, as illustrated in Figure 1(a). Using this analogy, the quantum emitter (QE) – which may be a quantum dot, nitrogen-vacancy (NV) center, or exciton in 2D materials – represents the fixed ball, as its material properties often constrain its positioning due to the complexities of synthesis and fabrication. Conversely, tuning the cavity system is more feasible, as its modes and couplings can be designed and engineered using readily available materials and established fabrication techniques. This tunable cavity system acts as the adjustable net, designed to hybridize effectively with the fixed QE.

Our approach involves developing an SSL system, acting as a tailored net. This concept is exemplified by optoplasmonic systems that combine plasmonic antennas with photonic cavities. In these systems, a spatial-localized mode offered by plasmonic antennas is engineered to concentrate the electromagnetic field within a small mode volume, which enhances coupling with the QE, effectively “capturing” the ball. Concurrently, a spectral-localized mode (*e*.*g*., photonic cavities) with a high Q-factor can be designed to preserve coherence and extend the lifetime of the QE.

Indeed, such a QE-SSL hybridization is crucial for quantum applications involving QEs. For example, (i) Quasi-bound State (qBS) [24]: An exotic eigenstate in the QE-SSL system that protects the quantum coherence of the QE. (ii) Exceptional Point (EP) Control [38]: Spatio-spectral couplings are adjusted to reach the EP, enhancing interaction between the QE and the spatial-localized mode. (iii) Single Photon Source (SPS) [39]: The SSL system spatially captures the QE, enhancing emission rates with the spatial-localized mode, while the spectral-localized mode maintains the indistinguishability and purity of emitted photons. (iv) Entanglement [8]: Strong coupling among QEs occurs under ambient conditions, with spatial-localized modes amplifying QE oscillations and spectral-localized modes preserving coherence, thereby enhancing overall concurrence.

### 2.1 QE-SSL hybridization

The QE-SSL hybridization can be theoretically captured on the basis of a cQED framework, where an SSL system exhibits spatio-spectral modal coupling, described by a generalized three-body quantum model. Here, we consider a simplified system and model with one spatial-localized mode *α* and one spectral-localized mode *β*, characterized by resonant frequencies *ω*_*α*_ and *ω*_*β*_, and decay rates *γ*_*α*_ and *γ*_*β*_ respectively, with the assumption that *γ*_*α*_ ≫ *γ*_*β*_. The QE is modeled as a two-level system with a transition dipole moment *μ*_e_ aligned with the local electric field and a transition frequency *ω*_e_. Its intrinsic decay rate is calculated as $\gamma_{\text{e}}=\omega_{\text{e}}^{3}\mu_{\text{e}}^{3}/\left(3\pi \epsilon_{0}\hbar c^{3}\right)$ , where *ϵ*_0_ is the vacuum permittivity and *c* the speed of light in a vacuum. In this work, we choose the fixed “ball”, the QE, characterized by the dipole moment *μ*_e_ = 46 *D* and *ω*_e_ = 1.85 eV (670 nm), as indicated in Figure 1(b) (red). This selection is based on the properties of in-plane excitons in monolayer MoS_2_ [40], with the QE characteristics obtained from a previous work [41], which is considered a promising candidate for future quantum devices [42], [43].

The system is described in the Fock state representation, with a full Hamiltonian under the rotating-wave approximation, which is expressed as:

(1) $$H=H_{0}+H_{\text{I}}+H_{\text{d}},$$

including the free Hamiltonian:

(2) $$H_{0}=\left(\omega_{\alpha }-\omega \right){\hat{\alpha }}^{†}\hat{\alpha }+\left(\omega_{\beta }-\omega \right){\hat{\beta }}^{†}\hat{\beta }+\left(\omega_{\text{e}}-\omega \right){\hat{\sigma }}^{+}{\hat{\sigma }}^{-},$$

the interaction Hamiltonian:

(3) $$\begin{array}{ll} H_{\text{I}} & =g_{\alpha \beta }\left({\hat{\alpha }}^{†}\hat{\beta }+{\hat{\beta }}^{†}\hat{\alpha }\right)+g_{\text{e}\alpha }\left({\hat{\sigma }}^{+}\hat{\alpha }+{\hat{\alpha }}^{†}{\hat{\sigma }}^{-}\right)\\ & \;+g_{\text{e}\beta }\left({\hat{\sigma }}^{+}\hat{\beta }+{\hat{\beta }}^{†}{\hat{\sigma }}^{-}\right), \end{array}$$

and the driving Hamiltonian:

(4) $$H_{\text{d}}=\Omega_{\alpha }\left({\hat{\alpha }}^{†}+\hat{\alpha }\right)+\Omega_{\beta }\left({\hat{\beta }}^{†}+\hat{\beta }\right)+\Omega_{\text{e}}\left({\hat{\sigma }}^{+}+{\hat{\sigma }}^{-}\right),$$

where *ω* denotes the probing frequency. The operators ${\hat{\alpha }}^{†}$ and $\hat{\alpha }$ represent the creation and annihilation operators for mode *α*, while and ${\hat{\beta }}^{†}\left(\hat{\beta }\right)$ do the same for the mode *β*. The QE is characterized by transition operators ${\hat{\sigma }}^{+}=|e〉〈g|$ and ${\hat{\sigma }}^{-}=|g〉〈e|$ , where |*e*⟩ and |*g*⟩ correspond to the excited and ground states, respectively. The interaction Hamiltonian *H*_I_ encapsulates the interactions in the system, including the *α* − *β* modal coupling and the QE coupling with both modes, denoted by the coupling rates *g*_*αβ*_, *g*_e*α*_ and *g*_e*β*_. The driving Hamiltonian *H*_d_ describes the external field pumping, where Ω_*α*_, Ω_*β*_ and Ω_e_ represent the coherent pumping amplitudes [44] for exciting modes *α*, *β* and the QE, respectively. For simplicity, we set *ℏ* = 1 in the derivations.

We calculate the steady-state density matrix *ρ*(*ω*) at the probing frequency *ω*, and the spectral intensity for *α*, *β*, and the QE is given by: $I_{i}\left(\omega \right)=\left\langle {\hat{O}}^{†}\hat{O}\right\rangle =\mathrm{Tr}\left[{\hat{O}}^{†}\hat{O}\rho \left(\omega \right)\right]$ , where *i* = *α*, *β*, *e* and $\hat{O}$ represents $\hat{\alpha }$ , $\hat{\beta }$ , and ${\hat{\sigma }}^{-}$ , respectively. These operators characterize the spectral response of different components in the system [44], [45]. To capture both the strong modal splitting and spectral effects induced by the QE, we plot the normalized spectral intensity of mode *α*, *I*_*α*_(*ω*), in Figure 1(b). As illustrated, the QE is fixed, analogous to a stationary ball, with a narrow line shape resulting from its intrinsic spontaneous emission rates, typically around 1 μeV [46], [47]. The SSL system represented as a tailored net, is treated as a “zero-detuned system” comprising both the spatial-localized mode and the spectral-localized mode, which share the same resonance energy defined as *ω*_SSL_ = *ω*_*α*_ = *ω*_*β*_. The dashed lines in the spectra represent the strong modal coupling when no interaction occurs with the QE, resulting in two symmetrically split peaks around *ω*_SSL_. We refer to this line shape as “SSL splitting”, analogous to the Rabi splitting observed in strong coupling between a QE and a cavity [7], [48], [49]. The SSL system is characterized by the QE-SSL detuning defined as Δ_SSL_ = *ω*_SSL_ − *ω*_e_. This relationship corresponds to: Δ_SSL_ < 0 (QE aligns with the right SSL peak); Δ_SSL_ = 0 (QE aligns with the middle valley); Δ_SSL_ < 0 (QE aligns with the left SSL peak). This design enables customized interactions between the QE and the SSL system. These interactions modify the SSL splitting spectra (blue-shaded) and induce spectral hole-burning (SHB) phenomena. Originally introduced in the context of laser-induced bleaching [45], [50], SHB also describes the destructive “holes” that appear in the spectrum [22], [51]. As illustrated, the QE creates holes in the SSL peaks: to the right (Δ_SSL_ < 0) and the left (Δ_SSL_ < 0), while the case of Δ_SSL_ = 0 shows an enlargement of the central hole in the SSL splitting. This enlargement suggests that the SSL system effectively captures the QE spatially and spectrally. A time-domain analysis is essential to uncover the underlying mechanisms.

### 2.2 Quasi-bound-state in a QE-SSL system

The time evolution of the QE-SSL system is described based on a master equation:

(5) $$\frac{\partial \rho }{\partial t}=i\left[\rho ,H\right]+\frac{\gamma_{\alpha }}{2}L\left[\hat{\alpha }\right]\rho +\frac{\gamma_{\beta }}{2}L\left[\hat{\beta }\right]\rho +\frac{\gamma_{\text{e}}}{2}L\left[{\hat{\sigma }}^{-}\right]\rho ,$$

where the dynamics of the density matrix *ρ* is governed by coherent evolution under the Hamiltonian *H* and losses associated with the modes *α*, *β*, and the QE. These losses are described by Lindblad terms in the standard form: $L\left[\hat{O}\right]\rho =2\hat{O}\rho {\hat{O}}^{†}-{\hat{O}}^{†}\hat{O}\rho -\rho {\hat{O}}^{†}\hat{O}$ , where $\hat{O}$ can represent $\hat{\alpha }$ , $\hat{\beta }$ or ${\hat{\sigma }}^{-}$ .

The respective equations of motion can be expressed as:

(6) $$\begin{array}{c} 〈\dot{\hat{\alpha }}〉=-\left(\gamma_{\alpha }/2+i\omega_{\alpha }\right)〈\hat{\alpha }〉-ig_{\text{e}\alpha }〈{\hat{\sigma }}^{-}〉-ig_{\alpha \beta }〈\hat{\beta }〉,\\ 〈\dot{\hat{\beta }}〉=-\left(\gamma_{\beta }/2+i\omega_{\beta }\right)〈\hat{\beta }〉-ig_{\text{e}\beta }〈{\hat{\sigma }}^{-}〉-ig_{\alpha \beta }〈\hat{\alpha }〉,\\ 〈{\dot{\hat{\sigma }}}^{-}〉=-\left(\gamma_{\text{e}}/2+i\omega_{\text{e}}\right)〈{\hat{\sigma }}^{-}〉-ig_{\text{e}\alpha }〈\hat{\alpha }〉-ig_{\text{e}\beta }〈\hat{\beta }〉. \end{array}$$

These equations indicate the effective non-Hermitian Hamiltonian in the single-excitation subspace, which can be expressed as follows:

(7) $$H_{\text{eff}}=\left[\begin{array}{ccc} \Delta_{\text{SSL}}-i\gamma_{\alpha }/2 & g_{\alpha \beta } & g_{\text{e}\alpha }\\ g_{\alpha \beta } & \Delta_{\text{SSL}}-i\gamma_{\beta }/2 & g_{\text{e}\beta }\\ g_{\text{e}\alpha } & g_{\text{e}\beta } & -i\gamma_{\text{e}}/2 \end{array}\right].$$

This Hamiltonian possesses three eigenvalues, one of which is purely real when the QE-SSL detuning is given by:

(8) $$\Delta_{\text{SSL}}^{\text{qBS}}=\omega_{\text{SSL}}-\omega_{\text{e}}=g_{\text{e}\beta }\left(\frac{g_{\alpha \beta }}{g_{\text{e}\alpha }}-\frac{g_{\text{e}\alpha }}{g_{\alpha \beta }}\right).$$

The corresponding eigenstate acts as a “bound state” that protects the quantum coherence of the QE. Although achieving ideal protection with zero loss is challenging due to intrinsic decay rates *γ*_e_, *γ*_*α*_ and *γ*_*β*_, this eigenstate still results in the slowest decay of the QE, thus being referred to as qBS [24]. Under a special condition of *g*_e*β*_ = 0, the qBS is positioned at $\Delta_{\text{SSL}}^{\text{qBS}}=0$ , indicating that the SSL is in resonance with the QE. In Figure 1(c), the emitter dynamics represent the time evolution of the QE’s excited-state population, plotted on a logarithmic scale as $log\left\langle {\hat{\sigma }}^{+}{\hat{\sigma }}^{-}\right\rangle \left(t\right)$ . These dynamics show the decay of the QE from its excited state, providing clear evidence of quantum coherence protection for Net ②, which is resonant with the QE $\left(\Delta_{\text{SSL}}=\Delta_{\text{SSL}}^{\text{qBS}}=0\right)$ and forms the qBS.

## 3 Designing a full-plasmonic SSL system

**Figure 2:**
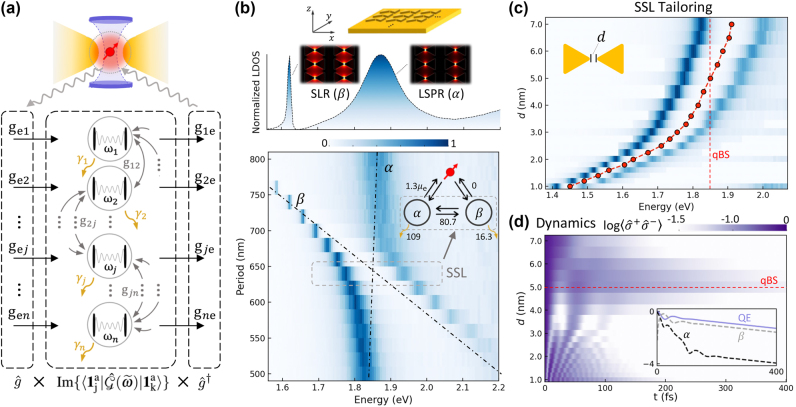
Designing a full-plasmonic tunable SSL system. (a) Quantum modal-coupling model for extracting coupling rates from the response field intensity under a point dipole source excitation. (b) Plasmonic bowtie-array metasurface showcasing localized surface plasmon resonance (LSPR) mode (*α*, spatially localized mode, *ω*_*α*_ = 1.87 eV, *γ*_*α*_ = 117 meV) and surface lattice resonance (SLR) mode (*β*, spectrally localized mode, *ω*_*β*_ = 1.62 eV, *γ*_*β*_ = 10.6 meV) before modal coupling. The calculated normalized local-density-of-states (LDOS) at the position of the dipole source displays an anti-crossing feature as the lattice period along the *x*-axis varies from 500 to 800 nm, with observed spectral splitting at the crossing point (*ω*_*α*_ = *ω*_*β*_) indicating a zero-detuned SSL system (extracted *g*_*αβ*_ = 80.7 meV) with strong modal coupling. (c) Tailoring zero-detuned SSL systems: the splitting spectrum is tuned as *d* increases from 1.0 to 7.0 nm, corresponding to tunable *ω*_SSL_ (red dots) from 1.45 to 1.91 eV (650–855 nm). (d) For a fixed QE with *ω*_e_ = 1.85 eV and *μ*_e_ = 46 *D* throughout this study, we can select an SSL system from the map (c) to achieve the qBS at *d* = 5 nm with Δ_SSL_ = *ω*_SSL_ − *ω*_e_ = 0 when the dynamics of QE is traced. At the qBS, full dynamics of *α*, *β*, and the QE are shown in the inset on a logarithmic scale.

Integrating and aligning plasmonic antennas with photonic cavities in optoplasmonic systems can be challenging. We propose using high-Q surface lattice resonances (SLR) within an array architecture as the spectral localization component, as shown in Figure 2. This method simplifies integration with plasmonic nanoantennas, creating an all-plasmonic platform. Introducing a QE from monolayer MoS_2_ into this platform can lead to the formation of the qBS. The system allows for straightforward tuning to optimize the interaction with the QE. In this scenario, we assume that the spatial-localized mode *α* and the QE are resonantly driven by a weak pump, such that Ω_*α*_ = Ω_e_ ∼ 0.1*γ*_e_.

### 3.1 Modal-coupling model

Modal coupling between two modes – one spatially localized with a broad band and the other spectrally localized with a narrow band – typically results in Fano line shapes [52], which depend on the modes’ properties and their coupling. To evaluate system performance in the quantum regime and differentiate near-field coupling from far-field interference, we previously developed a model that analyzes Fano line shapes from scattering by extracting parameters like coupling and decay rates (radiative and non-radiative) [51], rather than relying on shape parameters [53]. This approach provides a more physically grounded description and enables analysis of coupling across multiple modes. In this work, we extend this by developing a modal-coupling model to analyze Fano line shapes generated by fields excited from a point source.

In a modal-coupling system, the point source is modeled as a dipole placed at *r*_e_, where the field generated by the point dipole simultaneously drives the dipole, as described by Fermi’s golden rule:

(9) $$\begin{array}{ll} \underset{f}{\sum }\Gamma_{i\to f} & =2\pi \underset{f}{\sum }|T_{i\to f}{|}^{2}\delta \left(E_{f}-E_{i}\right)\\ & \propto J\left(\omega \right). \end{array}$$

Here, $T_{i\to f}$ is the transition matrix that represents the probability of transition from the initial state |*i*⟩ to the final state |*f*⟩. *J*(*ω*) is the spectral density defined over the continuous spectrum [24], [54] and can also be expressed in terms of the LDOS, *ρ*(*r*_e_, *ω*) as:

(10) $$J\left(\omega \right)=\frac{\pi \omega }{6\epsilon_{0}}\rho \left(r_{\text{e}},\omega \right).$$

Equation (9) acts as a bridge connecting the classical and quantum frameworks, *i*.*e*., the total transition probabilities from a quantum perspective correspond to the spectral density over the continuous spectrum from the classical viewpoint, which can be computed using the Green’s function of the field [47] (see Supplementary Information S1 for detailed derivation).

In the quantum framework, transitions occur among photon states, moving from point source states to the states of the modal-coupling system and back to the point states, as illustrated in Figure 2(a). The modal-coupling system comprises *n* quasinormal modes (QNMs), where the *j*th (*j* = 1, 2, …, *n*) mode is defined as ${\tilde{\omega }}_{j}=\omega_{j}-i\gamma_{j}$ . Here, the real part *ω*_*j*_ represents the resonance frequency, while the imaginary part *γ*_*j*_ signifies the decay rate or loss associated with that mode. The *j*th and *k*th modes (*k* = 1, 2, …, *n* and *j* ≠ *k*) are coupled through the coupling rate *g*_*jk*_. These QNMs are considered discrete states of the modal-coupling system, where a photon transitions from the source states via coefficients *g*_e*j*_, representing the coupling strength between the QE at the point source and the *j*th mode, thereby highlighting their shared physical significance. Similarly, the photon returns to the source state via *g*_*j*e_. Due to the reciprocity of the system, we have $g_{\text{e}j}=g_{je}^{*}$ , which determines the transition matrix $T_{i\to f}$ . From Eq. (9), we have:

(11) $$\begin{array}{ll} \sum_{f}|T_{i\to f}{|}^{2}\delta \left(E_{f}-E_{i}\right) & =\sum_{j=1}^{n}g_{\text{e}j}\cdot \delta \left(\omega -\omega_{j}\right)\cdot g_{\text{e}j}^{*}\\ & =\frac{1}{\pi }\hat{g}\cdot \text{Im}\left\{\left\langle 1_{\text{j}}^{\text{a}}|\hat{G}\left(\tilde{\omega }\right)|1_{\text{k}}^{\text{a}}\right\rangle \right\}\cdot {\hat{g}}^{†}. \end{array}$$

The states $|1_{j}^{a}〉$ and $|1_{k}^{a}〉$ are defined within the system’s Hilbert space to derive the Green’s function $\hat{G}\left(\tilde{\omega }\right)$ in the context of the Schrödinger equation for this system. The term $\left\langle 1_{j}^{a}|\hat{G}\left(\tilde{\omega }\right)|1_{k}^{a}\right\rangle$ is evaluated using perturbation theory [55], resulting in the reciprocal of a non-Hermitian matrix:

(12) $${\left[\begin{array}{ccc} \tilde{\omega }-\omega_{1}+\frac{i}{2}\gamma_{1} & \ldots & -g_{1n}\\ \vdots & \ddots & \vdots \\ -g_{n1} & \ldots & \tilde{\omega }-\omega_{n}+\frac{i}{2}\gamma_{n} \end{array}\right]}^{-1}.$$

The vector $\hat{g}=\left[g_{\text{e}1},\ldots ,g_{\text{e}n}\right]$ represents the transitions between the point source and the modal-coupling system.

By combining Eqs. (9)–(12), we derive the LDOS relationship (see Supplementary Information S2 for details):

(13) $$\begin{array}{ll} & {\left[\begin{array}{c} g_{\text{e}1}\\ \vdots \\ g_{\text{e}n} \end{array}\right]}^{\text{T}}Im{\left[\begin{array}{ccc} \omega -\omega_{1}+\frac{i}{2}\gamma_{1} & \ldots & -g_{1n}\\ \vdots & \ddots & \vdots \\ -g_{n1} & \ldots & \omega -\omega_{n}+\frac{i}{2}\gamma_{n} \end{array}\right]}^{-1}\left[\begin{array}{c} g_{\text{e}1}\\ \vdots \\ g_{\text{e}n} \end{array}\right]\\ & \;\propto \frac{\pi \omega }{6\epsilon_{0}}\rho \left(r_{\text{e}},\omega \right). \end{array}$$

This relationship takes the same form as those reported in previous works [54]. To extract *g*_e*j*_, *g*_*jk*_, *ω*_*j*_ and *γ*_*j*_, we fit the quantum framework described in Eq. (13) to the classical results of LDOS, which are typically calculated through numerical simulations. The extracted mode properties and coupling parameters will serve as inputs for our three-body quantum model described in Sections 2.1 and 2.2. In the subsequent section, this fitting process will be illustrated in the context of designing a plasmonic bowtie-array metasurface.

### 3.2 Numerical designing of plasmonic bowtie-array metasurfaces

As shown in Figure 2(b), our SSL system features a gold bowtie array on a gold substrate, creating a plasmonic metasurface. The bowties are arranged in an orthogonal array along the directions of *x* and *y*. This system exhibits two key modes. The spatial-localized mode *α*, corresponding to the localized surface plasmon resonance (LSPR), features a concentrated field at the gap’s center and oscillates in-plane along the *x* direction. The spectral-localized mode *β*, associated with surface lattice resonances (SLRs), results from the collective response of the LSPRs and is uniformly distributed over the surface [34], [35]. Under continuous excitation, the modes *α* and *β* coexist with the QE positioned at the center of the bowtie gap (see Supplementary Information Figure S1), enabling potential coupling among all three. We model this interaction using our generalized modal-coupling approach, which allows us to derive the coupling properties without making assumptions.

For any given bowtie geometry (*e*.*g*., *d* = 5.0 nm), we can adjust the array period along the *x* direction to achieve zero detuning between LSPR and SLR. To do so, we excite the structure with an electric dipole source (dipole moment aligned along the *x* direction) at the gap center on the surface and measure the normalized LDOS spectrum from the bowtie array. As shown in Figure 2(b), the LDOS spectra exhibit anti-crossing behavior as the period changes from 500 to 800 nm. This results in an SSL system at the crossing point, where *ω*_SSL_ = *ω*_*α*_ = *ω*_*β*_.

To conduct a quantitative analysis, we apply our modal-coupling model to extract coupling parameters by establishing the transition matrix for the *α* − *β* modal-coupling system according to Eq. (11). The LDOS spectrum is then calculated from the response electric field (see details in Supplementary Information S3), yielding the expression:

(14) $$\begin{array}{ll} & {\left[\begin{array}{c} g_{\text{e}\alpha }\\ g_{\text{e}\beta } \end{array}\right]}^{\text{T}}Im\left({\left[\begin{array}{cc} \omega -\omega_{\alpha }+\frac{i}{2}\gamma_{\alpha } & -g_{\alpha \beta }\\ -g_{\alpha \beta } & \omega -\omega_{\beta }+\frac{i}{2}\gamma_{\beta } \end{array}\right]}^{-1}\right)\left[\begin{array}{c} g_{\text{e}\alpha }\\ g_{\text{e}\beta } \end{array}\right]\\ & \;\propto \mu_{\text{e}}E_{\text{res}}^{x}\left(r_{\text{e}},\omega \right). \end{array}$$

This equation enables us to fit the frequencies *ω*_*α*_ and *ω*_*β*_, decay rates *γ*_*α*_ and *γ*_*β*_, and coupling rates *g*_*αβ*_, *g*_e*α*_, and *g*_e*β*_ to the response electric field $E_{\text{res}}^{x}\left(r_{\text{e}},\omega \right)$ in the *x* direction obtained from numerical simulations.

We analyze the excited LDOS for an LSPR-SLR metasurface with a large period of 740 nm, revealing distinct linear spectra for the *α* (LSPR) and *β* (SLR) modes, which do not couple (*i*.*e*., the superposition of Lorentzian shapes calculated with *ω*_*α*_ = 1.87 eV, *ω*_*β*_ = 1.62 eV, *γ*_*α*_ = 117 meV, and *γ*_*β*_ = 10.6 meV), as shown in Figure 2(b), top. As the period decreases, *ω*_*α*_ remains relatively stable, while *ω*_*β*_ shows significant variation, which can be fitted with linear trends [32], [36], [37]. At the crossing point for the resonant metasurface shown in Figure 2(b), bottom, we fit the data under the constraint *ω*_*α*_ ≈ *ω*_*β*_. We find *ω*_*α*_ = 1.86 eV and *ω*_*β*_ = 1.85 eV, with a small detuning of 0.01 eV due to discrete period selection at 20 nm intervals. This deviation is acceptable for the SSL system, allowing us to assume *ω*_*α*_ = *ω*_*β*_ in discussions where Δ_*αβ*_ ≤ 0.01 eV. The modal-coupling rate is found to be *g*_*αβ*_ = 80.7 meV, confirming strong modal coupling since *g*_*αβ*_ > (*γ*_*α*_ − *γ*_*β*_)/4 [47]. The coupling rate to the QE shows *g*_e*α*_ = 1.3*μ*_e_, consistent with previous findings [47]. Notably, the fitting indicates no coupling between the QE and *β* (see Supplementary Information S4). This may be explained by the SLR mechanism, where collective oscillations in the bowtie array overshadow any influence from the QE, which lacks sufficient strength to affect its neighbors [36]. In contrast, the LSPR acts as an antenna, amplifying the QE’s oscillation and facilitating interaction with neighboring bowties.

Next, we can repeat this process (numerical simulation and fitting) by varying the bowtie geometry, specifically the inter-gap distance *d*, see Figure 2(c), to ensure that our SSL system frequency *ω*_SSL_ resonates with the fixed QE for reaching the qBS condition in Eq. (8). We observe an increase in *ω*_SSL_ from 1.45 eV to 1.91 eV as *d* varies from 1.0 nm to 7.0 nm.

We then input the extracted key parameters, *ω*_*α*(*β*)_, *γ*_*α*(*β*)_, *g*_e*α*_, *g*_e*β*_, and *g*_*αβ*_, into the master equation Eq. (5) of the three-body quantum model to study the dynamics. As an example, for a fixed QE with *ω*_e_ = 1.85 eV and *μ*_e_ = 46 *D*, the dynamics of the QE in the QE-SSL systems as a function of distance *d* are shown in Figure 2(d). The selected configuration shows the longest coherence lifetime under conditions for forming the qBS ( $\Delta_{\text{SSL}}=\Delta_{\text{SSL}}^{\text{qBS}}=0$ when *g*_e*β*_ = 0) according to Eq. (8). The corresponding SSL system has *d* = 5.0 nm and an optimized period of 640 nm. To illustrate how the qBS protects coherence, the dynamics of the QE, as well as the *α* and *β* modes, are shown in the Inset. With a dipole moment of 46 *D*, the QE satisfies the strong coupling condition with the lossy *α* mode. However, it decays along a trajectory similar to the long-lived *β* mode, which does not directly couple to the QE but is strongly coupled to *α*. This suggests that the *β* mode functions as a “back-end capacitor” in the three-body system, charged both directly through *α* → *β* and indirectly via QE → *α* → *β*. When *α* decays rapidly, *β* discharges to help preserve the QE’s coherence, effectively shielding it from the fast decay of the *α* mode.

In summary, we have developed a plasmonic bowtie-array metasurface that aligns with our objective of creating a simple, tunable SSL system, easily adaptable for constructing the desired QE-SSL systems. We have exemplified the qBS, which will be further explored in the next section to examine its properties in relation to the diversified scenarios of the QE-SSL hybridization.

## 4 Quantum coherence protection

To shed light on the intriguing dynamics of QE-SSL hybridization and the protection of quantum coherence, we explore the effects of various excitation pathways, the implications of breaking QE-SSL coupling symmetry, and the role of modal-coupling strength in this section. By examining these factors, we aim to uncover how they influence the interplay between the QE and the SSL system, enriching our understanding of their collaborative potential.

### 4.1 Effect of excitation pathways

**Figure 3:**
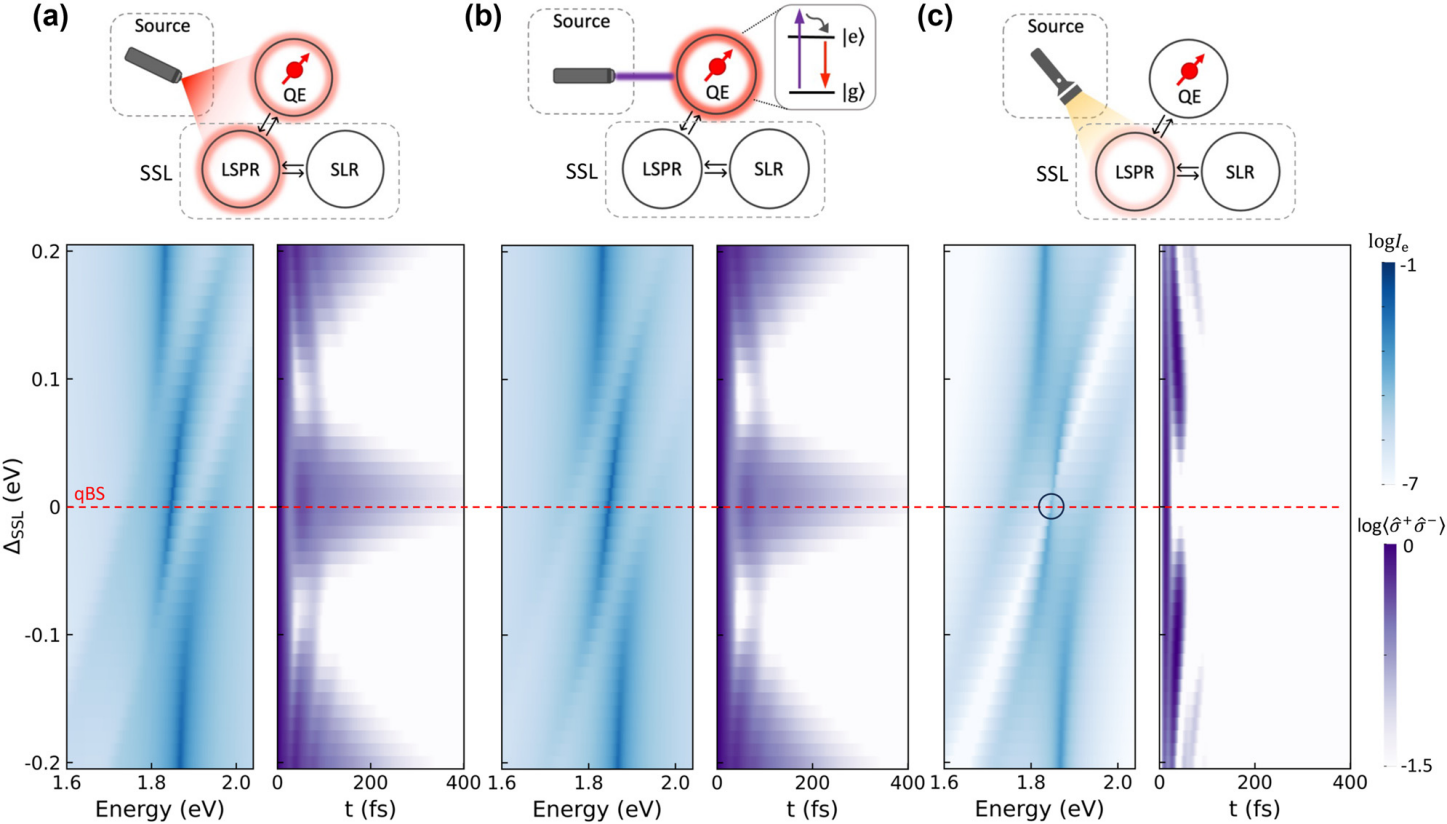
The effect of excitation pathways on quantum coherence protection. The dependence of the QE’s spectral intensity (blue) and dynamics (purple) on the pumping configurations: (a) resonant laser pumping to excite both QE and LSPR. (b) Higher-frequency laser pumping to excite the QE alone. (c) Ambient white-light pumping to excite the LSPR alone. Here, the QE is fixed with *ω*_e_ = 1.85 eV throughout this study, and the SSL systems are tuned with Δ_SSL_ = *ω*_SSL_ − *ω*_e_.

Different excitation conditions in experiments can affect the quantum coherence protection at the qBS. This section will compare various excitation pathways for the exemplified plasmonic bowtie metasurface SSL system, as illustrated in Figure 3. In the three-body quantum model described in Sections 2.1 and 2.2, the driving Hamiltonian interacts with different components – the QE, *α*, or *β* – serving as the excitation source to determine the steady-state spectral densities and establishes the initial excitation states of all components at the onset of the dynamics. The driving is assumed to be weakly coherent pumping, as described in Section 3.1 (Ω_*α*_ or Ω_e_ ∼ 0.1*γ*_e_) under the jump-free approximation [24], [56]. Note that Ω_*β*_ = 0 is set, as we assume *β* cannot be directly excited by an external source. For dynamics calculations, these weak pumps are insufficient to represent the dissipation trajectory. Instead, we initialize the system to its starting states using an ideal ultrafast pump pulse with negligible duration before the dynamics begin. Thus, the light sources discussed in this section, whether a laser or a halogen lamp [7], [41], are assumed to serve as a weak continuous pump for spectral intensity calculations and as an ideal ultrafast pump pulse to initialize the starting states for the dynamics calculation. They determine the different excitation pathways in the following three cases.

The first case (Figure 3(a)) represents “resonant excitation” [57], [58] , *i*.*e*., the default case throughout this study, where a laser with energy matching the QE irradiates the SSL-QE system. The broad linewidth of the *α* LSPR mode allows for simultaneous excitation with the QE, while the *β* SLR mode is not expected to be directly excited due to its formation mechanism. Dynamic calculations are performed with both *α* and the QE initially in their excited states, while *β* is in its ground state, as illustrated in the Inset dynamics of Figure 2(d). Now we assume the SSL system can be tuned freely, with Δ_SSL_ varying from −0.2 to 0.2 eV. The spectral intensity and dynamics of the QE are represented in two color maps using gradient shades of blue and purple. The spectral intensity reveals three deep blue arms, showing two anti-crossings at Δ_SSL_ = −0.08 and 0.08 eV, where the QE interacts with the left and right peaks of the SSL splitting (see Figure 1(b)). These arms indicate regions where the QE’s spectral intensity remains unaffected by the SSL system, correlating with prolonged quantum coherence time in the dynamic plot. Coherence time increases with greater detuning in the upper and lower regions, as the SSL system’s spectral range moves away from the QE. Conversely, coherence time decreases near the qBS at zero detuning, highlighted by a red dashed line, which marks the longest coherence time across the dynamic map. The anti-crossings in the spectral intensity map are symmetrically positioned around the qBS, and the dynamic map also displays a symmetric distribution centered at the qBS, establishing this QE-SSL system as symmetric with respect to detuning from the qBS.

The second case (Figure 3(b)) addresses “higher-frequency excitation”, where the laser frequency significantly exceeds the QE’s resonant frequency to avoid simultaneous excitation of the LSPR. In practical scenarios, the QE is not a pure two-level system: it often includes higher non-radiative states [59], [60], [61]. These higher states can be reached first, followed by non-radiative relaxation to the excited state |*e*⟩, allowing the QE to function as an initially excited two-level system within the SSL-QE framework. In this setting, the spectral intensity and dynamic maps show little variation from the first case in Figure 3(a), suggesting that simultaneous excitation of the LSPR does not significantly affect the QE’s performance in the QE-SSL system.

The third case (Figure 3(c)) explores an extraordinary method of driving the QE-SSL system using “ambient light” from conventional white light sources, such as halogen lamps [7], [41], commonly used in scattering measurements. These sources are spatially diffuse and spectrally broadband, which makes direct excitation of the QE challenging but effectively stimulates the LSPR [62], leveraging spatially localized modes with high decay rates across a broad spectrum. This results in distinct changes: the spectral intensity at the three arms decreases, and the prolonged coherence time seen in previous cases vanishes. Specifically, The qBS line (Δ_SSL_ = 0) intersects a white ribbon representing the SSL splitting valley, creating a vanishing point (highlighted by a black circle in Figure 3(c)) on the middle blue arm. This point corresponds to the shortest dynamics in the purple map. Here, symmetric dynamics in the upper and lower regions are shortened, indicating that the SSL system drives the QE through its SSL splitting peaks, accelerating decoherence. Notably, qBS cannot be achieved unless the QE is initially excited, highlighting the behavior of a dark state that is difficult to access through coupling pathways.

Despite these changes, symmetry about Δ_SSL_ = 0 in all maps is preserved, suggesting that this characteristic arises from the internal mechanisms of the QE-SSL system rather than the specific excitation pathways.

### 4.2 Breaking QE-SSL coupling symmetry

**Figure 4:**
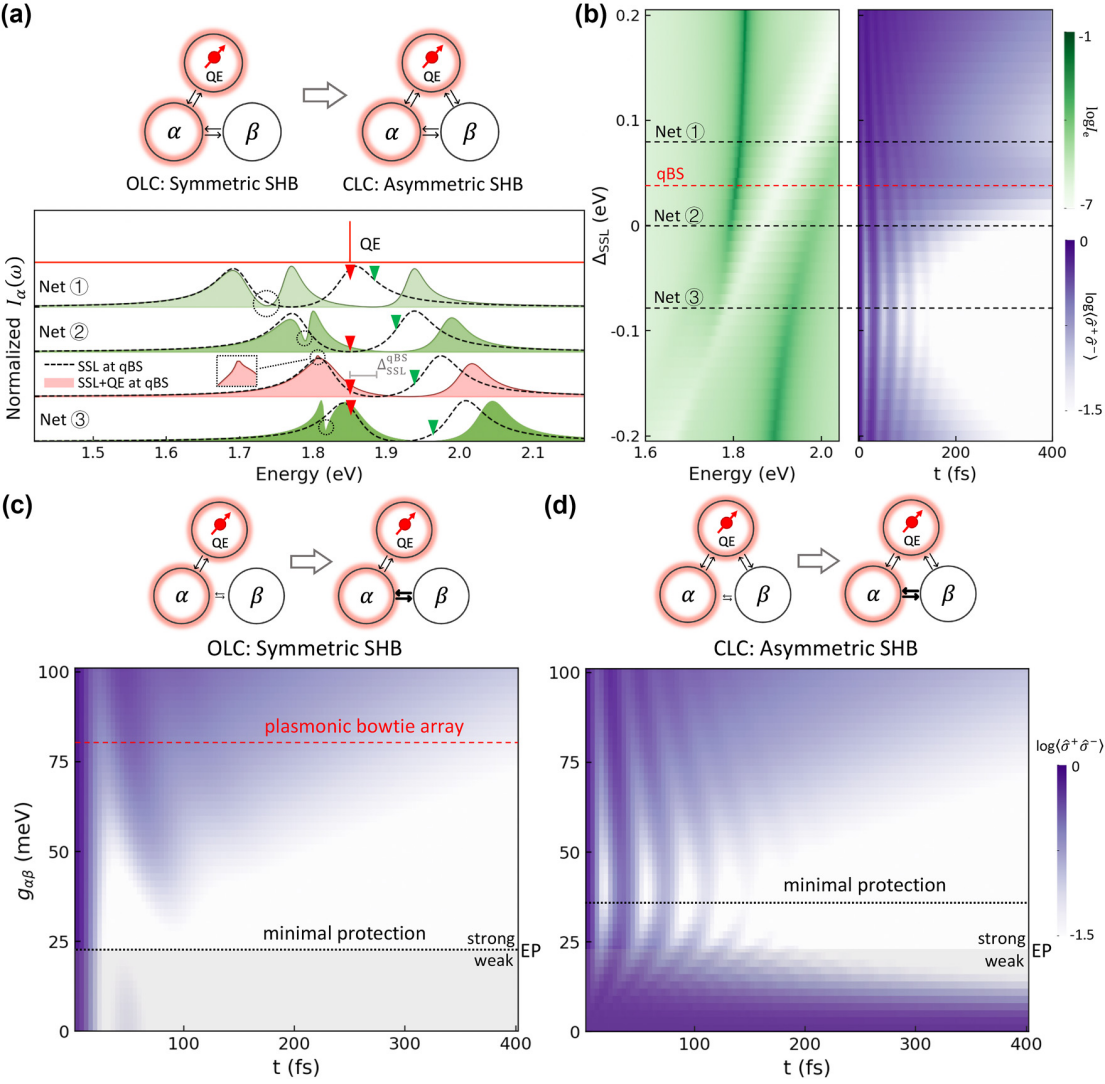
From open-loop coupling (OLC) symmetric to closed-loop coupling (CLC) asymmetric QE-SSL systems. (a) The same three SSL systems in Figure 1(b) (dashed lines), exhibiting SSL splitting, are tuned around a QE oscillating at *ω*_e_ = 1.85 eV. The QE (red triangles) induces asymmetric SHB features in the SSL-QE hybrid systems 1–3 (green-shaded) and a qBS hybrid system (pink-shaded) when *g*_e*β*_ = *g*_e*α*_ around right SSL peak (green triangles) and left SSL peak (circles). (b) For a fixed QE with *ω*_e_ = 1.85 eV and *μ*_e_ = 46 *D* throughout this study, we can design an SSL system to achieve the qBS according to Eq. (8). The dependence of spectral intensity (green) and dynamics (purple) on Δ_SSL_, where the four hybrid systems from (a) are labeled. (c) and (d) The dependence of dynamics on the modal-coupling strength *g*_*αβ*_ of *α* and *β* at qBS under different coupling conditions: (c) OLC *g*_e*β*_ = 0 and (d) CLC *g*_e*β*_ = *g*_e*α*_.

In previous case studies of plasmonic bowtie-array metasurfaces coupled to in-plane excitons in monolayer MoS_2_, the QE was only coupled to *α* – the spatial-localized LSPR mode as predicted by our modal-coupling model. However, the QE may also couple to the spectral-localized *β* mode in other QE-SSL systems. This section will differentiate between the two coupling scenarios, as shown in Figure 4.

As illustrated in Figure 4(a), we now assume *g*_e*β*_ = *g*_e*α*_, leading to closed-loop coupling (CLC) where all coupling links are established within the three-body quantum system. We present two additional scenarios in the Supplementary Information S5: *g*_*eβ*_ < *g*_*eα*_ and *g*_*eβ*_ > *g*_*eα*_. In contrast, the earlier system with *g*_e*β*_ = 0 features open-loop coupling (OLC). First, we present the SHB characteristics of the CLC, which is shown in green or pink in Figure 4(a). Unlike the OLC SHB in Figure 1(b), which displays symmetric spectral holes on the left and right SSL peaks as detuning varies, the CLC SHB demonstrates asymmetry. The asymmetry is evident when comparing the transitions from SSL splitting (dashed lines) to the QE-SSL (green-shaded) and qBS hybrid system (pink-shaded) spectra. The hole burnings by the QE (red triangles) are more pronounced on the right SSL peak, causing significant shifts and alterations in that region (green triangles). The SHB further splits the right SSL peak into left and right components, with the left component interacting with the left SSL peak, resulting in Fano line shapes, as indicated by the circles. This asymmetry aligns with findings from our previous work, where a microcavity mode created asymmetric holes in a plexcitonic system [22]. More excitingly, the qBS system exhibits a sharp tip atop the left SSL peak (Inset) instead of destructive line shapes. This occurs when the Fano asymmetry parameter *q* → ∞ [63], indicating the formation of a quasi-bound state in the continuum (quasi-BIC) within photonic structures [64].

The QE’s spectral intensity and dynamic maps for the asymmetric CLC are presented in Figure 4(b), revealing asymmetric characteristics, in contrast to Figure 3. In the spectral intensity map, the three color arms are reduced to two, with the upper arm covering a larger area and appearing stronger than the lower arm. The dynamic map retains three regions, but the middle region shifts upward. As predicted by Eq. (8), the qBS occurs Δ_SSL_ = 0.036 eV for *g*_e*β*_ = *g*_e*α*_, marked by the red dashed line, aligning very well with the dynamic simulation result. Thus, a non-zero *g*_e*β*_ establishes CLC among *α*, *β*, and the QE, resulting in symmetry breaking of SHB, spectral intensity, and dynamics, along with a shift in the qBS. Since the qBS remains robust even when symmetry is broken, asymmetric QE-SSL systems can be designed to protect coherence in situations where the symmetric coupling is challenging [21], [22].

### 4.3 Effect of modal-coupling strength

One interesting feature of our all-plasmonic resonant SSL is that it functions in a strong modal coupling regime where *g*_*αβ*_ > (*γ*_*α*_ − *γ*_*β*_)/4. We are interested in exploring the implications of the resonant SSL system operating outside this regime by comparing the coherence protection capability of the qBS against variations in *g*_*αβ*_ from 1 meV (weak modal coupling) to 100 meV (strong modal coupling), with the boundary at *g*_*αβ*_ = 23.2 meV. This comparison encompasses both the symmetric OLC system and the asymmetric CLC system. We highlight *g*_*αβ*_ = 80.7 meV, derived from our case study of plasmonic bowtie-array metasurface SSL system (*d* = 5.0 nm), in both cases using red dashed lines.

In the symmetric OLC system in Figure 4(c), the coherence time of the qBS decreases as *g*_*αβ*_ increases in the weak modal coupling regime but begins to improve in the strong modal coupling regime. This behavior is due to *β* acting as an additional loss in weak modal coupling and as a capacitor for coherence storage in strong modal coupling by an indirect pathway connecting with the QE through $QE\;\overset{g_{\text{e}\alpha }}{↔}\;\alpha \;\overset{g_{\alpha \beta }}{↔}\;\beta$ . As *g*_*αβ*_ increases further in the strong modal coupling regime, this storage capacity is enhanced. The boundary between weak and strong modal coupling, known as the exceptional point (EP), corresponds to the shortest coherence time of the qBS. This minimal protection of qBS is highlighted by a black dotted line. The finding aligns with our previous work [47], which identified the EP as the point of shortest QE lifetime in a plexcitonic system.

In the asymmetric CLC system shown in Figure 4(d), the qBS behavior varies significantly with *g*_*αβ*_. Compared to the OLC system, the CLC system exhibits more oscillations during qBS decay, although both show similar trends as *g*_*αβ*_ increases. The qBS achieves optimal protection in the weak modal coupling regime at *g*_*αβ*_ = 0, where the direct storage pathway $QE\;\overset{g_{\text{e}\beta }}{↔}\;\beta$ contributes to a longer lifetime. This effect weakens as the contribution from losses in *α* increase (*g*_*αβ*_ > 0) in the weak coupling regime. In the strong modal coupling regime, the indirect pathway $QE\;\overset{g_{\text{e}\alpha }}{↔}\;\alpha \;\overset{g_{\alpha \beta }}{↔}\;\beta$ strengthens, and both direct and indirect pathways work together, improving protection. Notably, the additional direct storage pathway in the CLC shifts the point of minimal protection away from the EP.

In summary, modal-coupling strength significantly affects qBS protection performance. In both symmetric and asymmetric systems, higher *g*_*αβ*_ enhances protection, with the asymmetric system offering better protection in the weak modal coupling regime. However, further increases in *g*_*αβ*_ may lead to improved qBS, indicating a positive outlook for developing stronger modal coupling in SSL systems.

## 5 Conclusions and outlook

This work highlights the potential of spatio-spectral localized (SSL) systems to enhance qubit manipulation and preserve coherence in quantum applications. By demonstrating the formation of a quasi-bound state (qBS) within a QE-SSL system, we gained insight related to the nature and relative importance of the interactions between a QE, a spatially localized mode, and a spectrally localized mode. For this, we developed a modal-coupling model that extracts the relevant quantum parameters from our design of a fully plasmonic tunable SSL system, *i*.*e*., plasmonic bowtie-array metasurface. This design enables precise alignment with a fixed QE at the qBS, enhancing coherence protection while reducing fabrication challenges. It can be easily tuned across an energy range of 1.45–1.91 eV by adjusting the period and gap thickness. Additionally, the design facilitates the straightforward integration of QEs from 2D materials, as these can be directly assembled onto the bowtie array [65]. The gold substrate elevates the localized surface plasmon resonance (LSPR) field at the bowtie tips, effectively capturing the QEs [66], [67]. Compared to optoplasmonic SSL systems, our plasmonic bowtie-array metasurface offers a simpler, more robust design with a single-layer fabrication process and potentially deterministic plexcitonic coupling at the single-emitter level. This is achieved by precisely positioning the emitter at the electromagnetic hotspot for optimal field enhancement and aligning its dipole moment with the mode polarization, ensuring consistent performance. Its seamless integration with 2D materials makes the system practical and scalable.

We also explored how the excitation pathway affects the performance of the QE-SSL system, emphasizing the importance of external excitation for forming a qBS. Our examination of the coupling symmetry between the QE and SSL, as well as the effects of modal-coupling strength, showed that the system’s SHB symmetry breaks when moving from open-loop to closed-loop coupling. Importantly, we found that stronger modal coupling enhances the protection of the qBS.

Further enhancing our understanding of QE-SSL interactions will enable exploring novel coupling strategies to optimize SSL system performance. Future research should focus on improving qubit coherence times and operational fidelity and assessing environmental impacts on SSL performance as well as experimental validation of our models and refinement of fabrication techniques. While our study demonstrates the interaction between a single QE and a single SSL system, the theory can be easily expanded to include multiple QEs and SSL systems by adjusting matrix dimensions. This expansion could lead to significant applications in plasmonic circuits. The ongoing exploration of SSL systems and their integration with quantum emitters will contribute to advancing robust quantum technologies, benefiting quantum computing, communication, and sensing.

## Supplementary Material

Supplementary Material Details
