# Supplementary material for: Spatio-spectral localized modal coupling for room-temperature quantum coherence protection
Source: Nanophotonics. 2025 Mar 20;14(7):885–98. doi: 10.1515/nanoph-2024-0574 (PMC11980875; doi:10.1515/nanoph-2024-0574)
Supplement: Supplementary file 1 — Supplementary Material Details [file j_nanoph-2024-0574_suppl_001.pdf]

Supplementary Information: Spatio-spectral localized modal coupling for room-temperature quantum coherence protection

Wen-jie Zhou[1,2], Yuwei Lu[3], Jingfeng Liu[4], Renming Liu[5], Lay Kee Ang[1], Ortwin Hess[6,*], Lin Wu[1,*]

[1] *Science, Mathematics, and Technology (SMT), Singapore University of Technology and Design, 8 Somapah Road, Singapore 487372.*

[2] *Department of Electrical and Computer Engineering, National University of Singapore, 4 Engineering Drive 3, Singapore 117583.*

[3] *Quantum Science Center of Guangdong–Hong Kong–Macao Greater Bay Area (Guangdong), Shenzhen, China 518045.*

[4] *College of Electronic Engineering and College of Artificial Intelligence, South China Agricultural University, Guangzhou, China 510642.*

[5] *School of Physics and Electronics, International Joint Research Laboratory of New Energy Materials and Devices of Henan Province, Henan University, Kaifeng, China 475004.*

[6] *School of Physics and CRANN Institute, Trinity College Dublin, Dublin 2, Ireland.*

Correspondence and requests for materials should be addressed to Lin Wu (email: lin_wu@sutd.edu.sg) and Ortwin Hess (email: ortwin.hess@tcd.ie)

(The Supporting Information comprises 14 pages, including 3 figures.)

S1. Modal-coupling field from classical to quantum

In this section, we provide a detailed derivation of how key parameters in quantum systems can be extracted from the practical measurement of field intensity. We begin by establishing the connection between classical and quantum models through the local density of states (LDOS), enabling the extraction of coupling parameters in the modal-coupling system.

Classical Perspective

From the classical perspective, an electric point dipole source oriented in a specific direction is placed at the position r_e of the QE to excite the modes. The response electric field is determined by solving the Helmholtz equation [1], given by:

$$\vec{E}_\mathrm{res}(r,\omega) = \frac{\vec{k}^2}{\varepsilon_0}\mathrm{Im}\vec{G}(r,r_\mathrm{e},\omega)\cdot\vec{\mu}(\omega), \tag{S1}$$

where $\mathrm{Im}\vec{G}$ is the imaginary part of the dyadic Green's function for the Helmholtz equation, $\vec{k} = \omega/c$ is the wave vector, and c is the speed of light in a vacuum. Assuming a constant dipole moment $\mu(\omega) = \mu_\mathrm{e}$, the LDOS at r_e can be computed using the Green's function [2]:

$$\rho(r_\mathrm{e},\omega) = \frac{6\mu_\mathrm{e}^2\omega}{\pi c^2}[\vec{e}_r\cdot\mathrm{Im}G(r_\mathrm{e},r_\mathrm{e},\omega)\cdot\vec{e}_r], \tag{S2}$$

where the dipole moment of the QE is defined as $\vec{\mu}(\omega) = \mu(\omega)\cdot\vec{e}_r$ and $\vec{e}_r$ is a unit vector in the direction of oscillation.

Quantum Perspective

The field generated by the point dipole simultaneously drives the QE's dipole, and the correspond-

ing transition rates are described by Fermi's golden rule:

$$\sum_f \Gamma_{i\to f} = 2\pi \sum_f |\mathcal{T}_{i\to f}|^2 \delta(E_f - E_i)$$
$$\propto J(\omega) \propto \rho(r_{\mathrm{e}}, \omega). \tag{S3}$$

Here, $\mathcal{T}_{i\to f}$ is the transition matrix that represents the probability of transition from the initial state $|i\rangle$ to the final state $|f\rangle$. The spectral density $J(\omega)$ [3,4] can be expressed in terms of the LDOS as:

$$J(\omega) = \frac{\pi\omega}{6\varepsilon_0}\rho(r_{\mathrm{e}}, \omega). \tag{S4}$$

Connection between Classical and Quantum Models

Eq. (S3) serves as a bridge between the classical and quantum frameworks. Specifically, the total transition probabilities in the quantum perspective correspond to the LDOS over the continuous spectrum in the classical framework, which can be computed using the Green's function of the field [2] by Eq. (S2).

S2. Modal-coupling theory

For a modal-coupling problem involving n quasi-normal modes (QNMs) excited by a point source, where $n \geq 2$, the total Hamiltonian is given by $\hat{H} = \hat{H}_0 + \hat{V}$. Here, $\hat{H}_0$ represents the free Hamiltonian, and $\hat{V}$ denotes the interaction potential. Both $\hat{H}_0$ and $\hat{V}$ are expressed in terms of Fock states as follows:

$$\hat{H}_0 = \sum_j^n \hbar\omega_j \hat{a}_j^\dagger \hat{a}_j + \sum_l \hbar\omega_l \hat{S}_l^\dagger \hat{S}_l + \sum_m \hbar\omega_m \hat{D}_m^\dagger \hat{D}_m, \tag{S5}$$

$$\hat{V} = \sum_j^n \sum_k^n \hbar g_{jk}(\hat{a}_j^\dagger \hat{a}_k + \hat{a}_k^\dagger \hat{a}_j) \tag{S6}$$

$$+ \sum_j^n \sum_l \hbar g_{lj}(\hat{S}_l^\dagger \hat{a}_j + \hat{a}_j^\dagger \hat{S}_l) + \sum_j^n \sum_m \hbar g_{mj}(\hat{D}_m^\dagger \hat{a}_j + \hat{a}_j^\dagger \hat{D}_m). \tag{S7}$$

Here, $\hat{a}_j$ (or $\hat{a}_j^\dagger$) denotes the annihilation (or creation) operator for the jth mode with resonance energy ω_j. Similarly, the point source and the loss reservoir are quantized as Fock states, labeled by integers l and m, corresponding to the system's excitation and energy leakage channels, respectively. $\hat{S}_l$ (or $\hat{S}_l^\dagger$) and $\hat{D}_m$ (or $\hat{D}_m^\dagger$) represents the annihilation (or creation) operators for the lth and m^{th} states.

The first term in $\hat{V}$ describes the near-field coupling between $\hat{a}_j$ and $\hat{a}_k$ with $j \neq k$, characterized by the coupling rate g_{jk}, with $g_{jk} = 0$ when $j = k$. The second term accounts for system excitation through coupling to the point source, described by the coupling rate g_{lj}. The second term addresses energy leakage via coupling to the loss reservoir, characterized by the coupling rate g_{mj}.

Based on Fermi's golden rule, the spectral density is derived by calculating the transition rate

between the initial (i) and final (f) states of the system. This involves evaluating the matrix element of the perturbing interaction between these states and integrating over the final state density, which accounts for the available final states and their distribution. The spectral density $J(\omega)$ follows the relationship:

$$J(\omega) \propto \sum_{i,f} |\mathcal{T}_{i\to f}|^2 \delta(E_f - E_i), \tag{S8}$$

where $\mathcal{T}_{i\to f}$ represents the transitions between the point source (inital state $|i\rangle$) and the modal-coupling system (final state $|f\rangle$). Thus, we have $|\mathcal{T}_{i\to f}|^2 = g_{\mathrm{e}j} \cdot g^*_{\mathrm{e}j}$ and the right-hand term of Eq. (S8) can be deduced as:

$$\begin{aligned} &\sum_{f} |\mathcal{T}_{i\to f}|^2 \delta(E_f - E_i) \\ &= \sum_{j=1}^{n} g_{\mathrm{e}j} \cdot \delta(\omega - \omega_j) \cdot g^*_{\mathrm{e}j} \\ &= \frac{1}{\pi} \hat{g} \cdot \mathrm{Im}\{\langle \mathbf{1}^{\mathrm{a}}_{\mathrm{j}} | \hat{\mathcal{G}}(\widetilde{\omega}) | \mathbf{1}^{\mathrm{a}}_{\mathrm{k}} \rangle\} \cdot \hat{g}^{\dagger}. \end{aligned} \tag{S9}$$

We use the Sokhotski–Plemelj theorem,

$$\lim_{\varepsilon\to 0^+} \frac{1}{x + i\varepsilon} = i\pi\delta(x) + \mathcal{P}(1/x), \tag{S10}$$

where $\mathcal{P}$ denotes the Cauchy principal value, to treat the delta function as the imaginary part of $\langle \mathbf{1}^a_j | \hat{\mathcal{G}}(\widetilde{\omega}) | \mathbf{1}^a_k \rangle$. The states $|\mathbf{1}^a_j\rangle$ and $|\mathbf{1}^a_k\rangle$ are defined within the system's Hilbert space to derive the Green's function $\hat{\mathcal{G}}(\widetilde{\omega})$ in the context of the Schrödinger equation for the system. The operator $\hat{g}$ represents a transtion vector $[g_{\mathrm{e}1}, \cdots, g_{\mathrm{e}n}]$, under the assumption that each mode has distinct excitation channels from the QE.

To determine the middle term $\langle \mathbf{1}^a_j | \hat{\mathcal{G}}(\widetilde{\omega}) | \mathbf{1}^a_k \rangle$, where $\hat{\mathcal{G}}(\widetilde{\omega})$ satisfies the equation $(\widetilde{\omega} - \hat{H}_0 - \hat{V})\hat{\mathcal{G}}(\widetilde{\omega}) = 1$, we treat the system potential $\hat{V}$ as a perturbation of the free Hamiltonian $\hat{H}_0$. The

reciprocal of Green's function is expanded [5] as:

$$\hat{\mathcal{G}}(\widetilde{\omega})^{-1} = \widetilde{\omega} - \hat{H}_0 - \hat{R}(\widetilde{\omega}) = \widetilde{\omega} - \hat{H}_0 - [\hat{V} + \hat{V}\frac{\hat{Q}}{\widetilde{\omega} - \hat{H}_0}\hat{V} + \hat{V}\frac{\hat{Q}}{\widetilde{\omega} - \hat{H}_0}\hat{V}\frac{\hat{Q}}{\widetilde{\omega} - \hat{H}_0}\hat{V} + \cdots], \quad \text{(S11)}$$

where $\hat{Q} = \hat{Q}^{\mathrm{e}} + \hat{Q}^{\mathrm{d}} = \sum_l |\mathbf{1}_l^{\mathrm{e}}\rangle\langle\mathbf{1}_l^{\mathrm{e}}| + \sum_m |\mathbf{1}_m^{\mathrm{d}}\rangle\langle\mathbf{1}_m^{\mathrm{d}}|$ represents the reservoir Hilbert space, with $\hat{P} + \hat{Q} = \mathbb{1}$. For diagonal elements ($j = k$),

$$[\langle\mathbf{1}_j^a|\hat{\mathcal{G}}(\widetilde{\omega})|\mathbf{1}_j^a\rangle]^{-1} = \widetilde{\omega} - \langle\mathbf{1}_j^a|\hat{H}_0|\mathbf{1}_j^a\rangle - \langle\mathbf{1}_j^a|\hat{R}(\widetilde{\omega})|\mathbf{1}_j^a\rangle = \widetilde{\omega} - \omega_j - \frac{i}{2}\gamma_j, \quad \text{(S12)}$$

and for non-diagonal elements ($j \neq k$):

$$[\langle\mathbf{1}_j^a|\hat{\mathcal{G}}(\widetilde{\omega})|\mathbf{1}_k^a\rangle]^{-1} = 0 - \langle\mathbf{1}_j^a|\hat{H}_0|\mathbf{1}_k^a\rangle - \langle\mathbf{1}_j^a|\hat{R}(\widetilde{\omega})|\mathbf{1}_k^a\rangle = -g_{jk}. \quad \text{(S13)}$$

Substitute Eq. (S12) and Eq. (S13) to Eq. (S9), the general form of the spectral density $J(\omega)$ is then given by:

$$J(\omega) \propto \frac{1}{\pi}\begin{bmatrix} g_{\mathrm{e}1} \\ \vdots \\ g_{\mathrm{e}n} \end{bmatrix}^{\mathrm{T}} \mathrm{Im}\left(\begin{bmatrix} \omega - \omega_1 + \frac{i}{2}\gamma_1 & \cdots & -g_{1n} \\ \vdots & \ddots & \vdots \\ -g_{n1} & \cdots & \omega - \omega_n + \frac{i}{2}\gamma_n \end{bmatrix}^{-1}\right)\begin{bmatrix} g_{\mathrm{e}1} \\ \vdots \\ g_{\mathrm{e}n} \end{bmatrix}, \quad \text{(S14)}$$

and the LDOS relationship is derived as:

$$\frac{\pi\omega}{6\varepsilon_0}\rho(r_{\mathrm{e}}, \omega) \propto \begin{bmatrix} g_{\mathrm{e}1} \\ \vdots \\ g_{\mathrm{e}n} \end{bmatrix}^{\mathrm{T}} \mathrm{Im}\left(\begin{bmatrix} \omega - \omega_1 + \frac{i}{2}\gamma_1 & \cdots & -g_{1n} \\ \vdots & \ddots & \vdots \\ -g_{n1} & \cdots & \omega - \omega_n + \frac{i}{2}\gamma_n \end{bmatrix}^{-1}\right)\begin{bmatrix} g_{\mathrm{e}1} \\ \vdots \\ g_{\mathrm{e}n} \end{bmatrix} \quad \text{(S15)}$$

S3. Full-wave simulation and extraction of key parameters for the SSL system

The Full-wave simulations for the plasmonic bowtie array are conducted using the FEM method with the COMSOL Multiphysics (RF module). The geometry settings are illustrated in Fig. S1: each bowtie consists of a dimer formed by two equilateral triangles, each with a side length of $w = 80$ nm and a height of $h = 30$ nm. The dimer is oriented along the x direction featuring a central gap with a thickness of d. All corners of the triangles are rounded with a radius of $r = 5$ nm. The bowties are arranged in an orthogonal lattice array using periodic boundary conditions with a constant period of 200 nm in the y direction, utilizing periodic boundary conditions. The array's overall periodicity is determined by the period in the x direction (named as "Period" in the main text). The bowtie array is placed on a substrate, which is used to engineer the hotspot in the gap when the LSPR mode is excited [6,7]. The entire structure is made of gold, with its permittivity described using the Drude-Lorentz function:

$$\varepsilon_{\mathrm{Au}}(\omega) = \varepsilon_{\mathrm{Au},\infty}\left(1 - \frac{\omega_{p,1}^2}{\omega^2 - \omega_{0,1}^2 + i\omega\Gamma_1} - \frac{\omega_{p,2}^2}{\omega^2 - \omega_{0,2}^2 + i\omega\Gamma_2}\right), \tag{S16}$$

where $\varepsilon_{\mathrm{Au},\infty} = 6$, $\omega_{p,1} = 5.37 \times 10^{15}$ rad/s, $\omega_{0,1} = 0$ and $\Gamma_1 = 6.22 \times 10^{13}$ rad/s; $\omega_{p,2} = 2.26 \times 10^{15}$ rad/s, $\omega_{0,2} = 4.57 \times 10^{15}$ rad/s and $\Gamma_2 = 1.33 \times 10^{15}$ rad/s [8]. The gold substrate elevates the hotspot to the upper surface of the entire structure, as indicated by the gray plane. This design facilitates the direct assembly of 2D materials on top [9], allowing for efficient coupling of their excitons with the LSPR. The QE is treated as a point source driving the structure, which, in our simulation, is modeled as an electric dipole source positioned at the center of the gap on the upper gray plane, where the QE is expected to be located. Under periodic boundary conditions, we effectively create

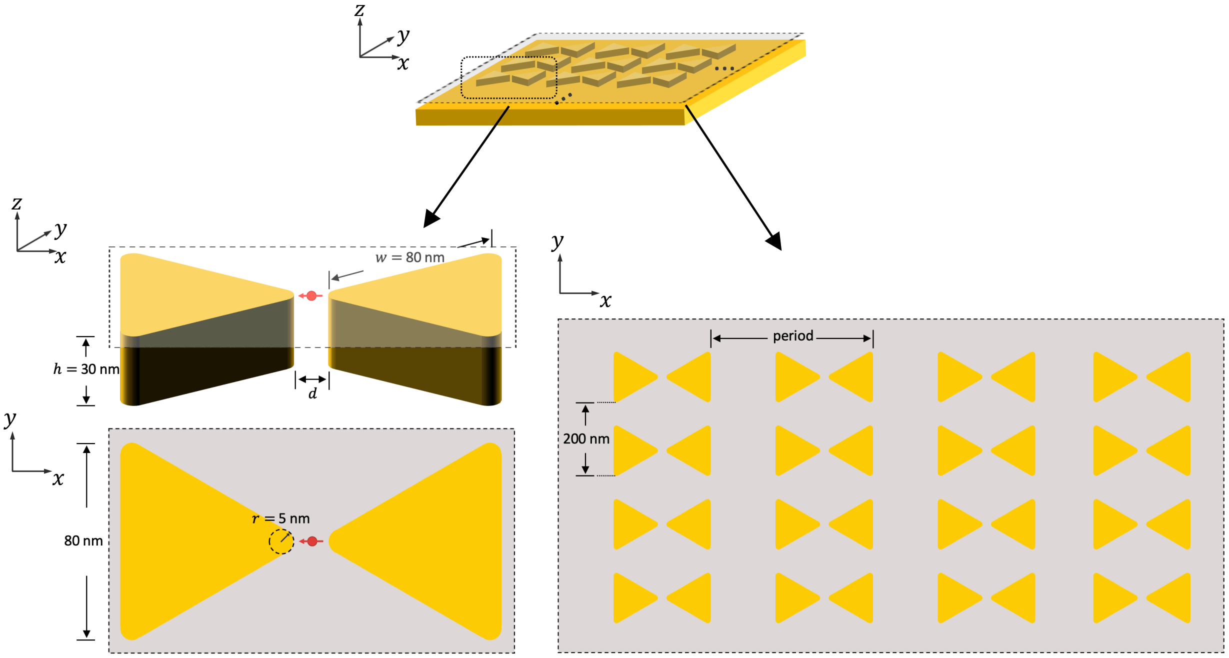

Figure S1: Geometric configuration of the plasmonic bowtie array. The gray plane represents the upper surface of the structure, where 2D materials can be assembled. A point dipole source is located at the center of each bowtie gap, designed to excite the SSL system and facilitate the extraction of quantum parameters.

an array of dipoles oscillating in unison. However, their near-field interactions can be neglected compared to their strong interactions with the corresponding bowties, as confirmed by the fitting result of $g_{e\beta} = 0$, indicating that the dipoles have no contribution to the SLR. Therefore, we can focus on a single dipole source and approximately ignore the effects of the array. The dipole is oriented in the x direction, aligning with the LSPR, with a dipole moment given by $\mu(\omega) = \varepsilon_0/\vec{k}^2$. Consequently, the response field in the x direction can be directly measured as the imaginary part of the field, given by $E^x_{\mathrm{res}}(r_\mathrm{e}, \omega) = \mathrm{Im}E^x(r_\mathrm{e}, \omega) = \mathrm{Im}G^x(r_\mathrm{e}, r_\mathrm{e}, \omega)$ at the same point of the dipole

source. The LDOS at this position is then calculated from the response field as follows:

$$\rho(r_\mathrm{e},\omega) = \frac{6\mu_\mathrm{e}^2\omega}{\pi c^2}\mathrm{Im}E^x(r_\mathrm{e},\omega), \tag{S17}$$

Fig. 2(b), top, shows the simulation result of the LDOS when the “Period” is set to 740 nm. Fig. 2(b), bottom, presents a series of simulation results with the “Period” varying from 500 nm to 800 nm in 20 nm intervals, plotted as a colormap in blue.

To extract the quantitative performance in quantum regime, the spectral density is given by

$$J(\omega) = \mu_\mathrm{e}E^x_\mathrm{res}(r_\mathrm{e},\omega) = \mu_\mathrm{e}\mathrm{Im}E^x(r_\mathrm{e},\omega). \tag{S18}$$

The fitting is performed using the equation in a simplified 2×2 form of Eq. (S15):

$$\begin{bmatrix} g_{\mathrm{e}\alpha} \\ g_{\mathrm{e}\beta} \end{bmatrix}^\mathrm{T} \mathrm{Im}\left(\begin{bmatrix} \omega-\omega_\alpha+\frac{i}{2}\gamma_\alpha & -g_{\alpha\beta} \\ -g_{\alpha\beta} & \omega-\omega_\beta+\frac{i}{2}\gamma_\beta \end{bmatrix}^{-1}\right)\begin{bmatrix} g_{\mathrm{e}\alpha} \\ g_{\mathrm{e}\beta} \end{bmatrix} \propto \mu_\mathrm{e}\mathrm{Im}E^x(r_\mathrm{e},\omega). \tag{S19}$$

S4. Modal coupling fittings

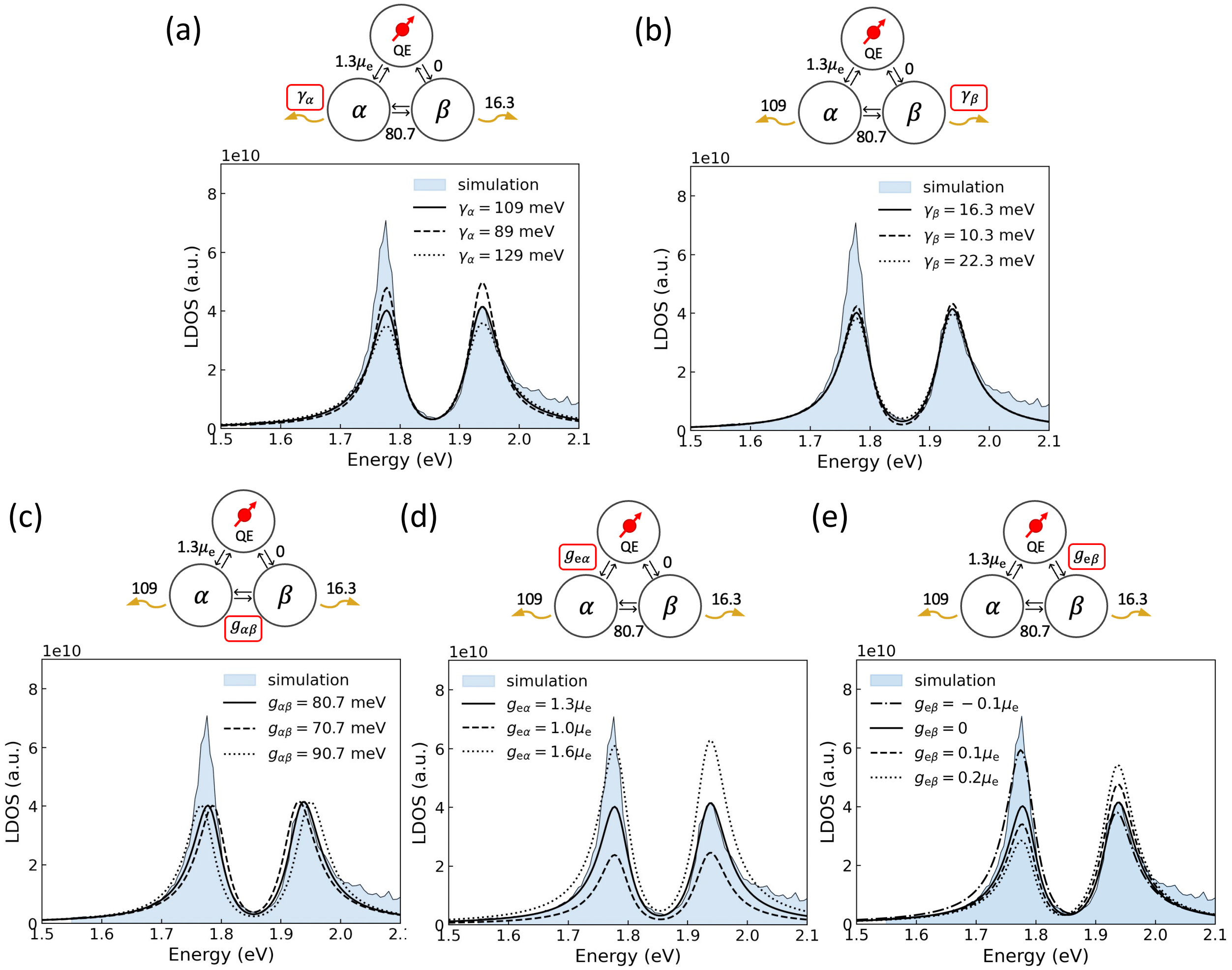

Figure S2: Modal coupling fittings by varying a single parameter.

Based on the SSL system geometry with a period of 640 nm and $d = 5$ nm, the fitting results are as follows: $\gamma_\alpha = 109$ meV, $\gamma_\beta = 16.3$ meV, $g_{\alpha\beta} = 80.7$ meV, $g_{e\alpha} = 1.3\mu_e$, and $g_{e\beta} = 0$. To assess the impact of varying one parameter (highlighted in red boxes) while keeping others constant, we consider the following scenarios as shown in Fig. S2: **(a)** Varying γ_α: Testing values of 99, 109 (original), and 119 meV, which results in simultaneous raising or lowering of both peaks. **(b)** Varying γ_β: Testing values of 10.3, 16.3 (original), and 22.3 meV, causing simultaneous

raising or lowering of both peaks. **(c)** Varying $g_{\alpha\beta}$: Testing values of 70.7, 80.7 (original), and 90.7 meV, which expands or contracts the valley of the splitting. **(d)** Varying $g_{\mathrm{e}\alpha}$: Testing values of $1.0\mu_{\mathrm{e}}$, $1.3\mu_{\mathrm{e}}$ (original), and $1.6\mu_{\mathrm{e}}$, leading to simultaneous raising or lowering of both peaks. **(e)** Varying $g_{\mathrm{e}\beta}$: Testing values of -$0.1\mu_{\mathrm{e}}$, 0 (original), $0.1\mu_{\mathrm{e}}$, and $0.2\mu_{\mathrm{e}}$, resulting in simultaneous lowering of the left peak and raising of the right peak for increased $g_{\mathrm{e}\beta}$.

Although a negative $g_{\mathrm{e}\beta} = -0.1\mu_{\mathrm{e}}$ improves the fit, the physical implications of a negative coupling rate are not well understood. Therefore, we assume $g_{\mathrm{e}\beta}$ to be non-negative in this work. Under this assumption, the optimal fit requires $g_{\mathrm{e}\beta} = 0$; otherwise, mismatches will occur in fitting both the left and right peaks. Notably, the fitting error for the left peak cannot be eliminated because the SLR mode exhibits a non-Lorentzian peak [10], while our modal-coupling model assumes it to be a QNM with a Lorentzian profile. As the quantization of collective SLR modes is not addressed, we aim to resolve this in future work to further improve our quantum framework.

S5. Closed-loop coupling to break symmetry

In this section, we present three scenarios to illustrate the asymmetry effect under CLC settings with different non-zero $g_{e\beta}$: (a) $g_{e\beta} = 0.3\mu_e < g_{e\alpha}$, (b) $g_{e\beta} = 1.3\mu_e = g_{e\alpha}$ (case in the main text), and (c) $g_{e\beta} = 2.3\mu_e > g_{e\alpha}$, as shown in Fig. S3.

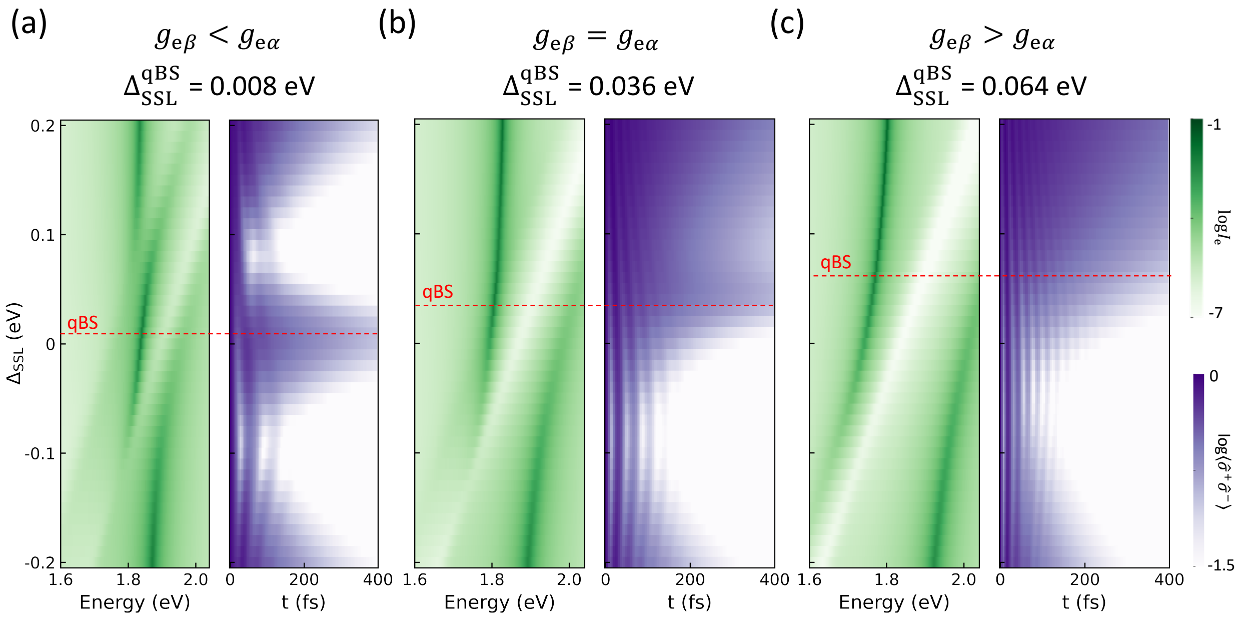

Figure S3: Closed-loop coupling (CLC) to break symmetry. Spectral intensity (green) and dynamics (purple) as functions of Δ_{SSL} for a closed-loop coupling (CLC) asymmetric QE-SSL system: (a) $g_{e\beta} = 0.3\mu_e < g_{e\alpha}$, (b) $g_{e\beta} = 1.3\mu_e = g_{e\alpha}$ (case in the main text), and (c) $g_{e\beta} = 2.3\mu_e > g_{e\alpha}$. The corresponding qBS positions are labeled in (a), (b), and (c).

The asymmetry in the system is minimal when $g_{e\beta} = 0.3\mu_e < g_{e\alpha}$, the quasi-bound state (qBS) is only slightly shifted by 0.008 eV from the center at $\Delta_{SSL} = 0$. However, as $g_{e\beta}$ increases,

the asymmetry becomes more pronounced, and the qBS shift grows correspondingly. This trend supports our conclusion that the CLC inherently introduces asymmetry, with the effect intensifying as $g_{e\beta}$ increases. All observed qBS shifts are consistent with our analytical formula in Eq. (8) of the main text: $\Delta_{\mathrm{SSL}}^{\mathrm{qBS}} = \omega_{\mathrm{SSL}} - \omega_{\mathrm{e}} = g_{\mathrm{e}\beta}\left(\frac{g_{\alpha\beta}}{g_{\mathrm{e}\alpha}} - \frac{g_{\mathrm{e}\alpha}}{g_{\alpha\beta}}\right)$. These findings underscore the crucial role of coupling strength in driving asymmetry and influencing the qBS shift in the system.

Reference

1. Novotny, L. & Hecht, B. *Principles of nano-optics* (Cambridge university press, 2012).

2. Zhou, W. *et al.* Exceptional points unveiling quantum limit of fluorescence rates in non-hermitian plexcitonic single-photon sources. *APL Quantum* **1** (2024).

3. Medina, I., García-Vidal, F. J., Fernández-Domínguez, A. I. & Feist, J. Few-mode field quantization of arbitrary electromagnetic spectral densities. *Physical Review Letters* **126**, 093601 (2021).

4. Lu, Y.-W. *et al.* Unveiling atom-photon quasi-bound states in hybrid plasmonic-photonic cavity. *Nanophotonics* **11**, 3307–3317 (2022).

5. Cohen-Tannoudji, C., Dupont-Roc, J. & Grynberg, G. *Atom-photon interactions: basic processes and applications* (John Wiley & Sons, 1998).

6. Xiong, X. *et al.* Control of plexcitonic strong coupling via substrate-mediated hotspot nano-engineering. *Advanced Optical Materials* **10**, 2200557 (2022).

7. Xiong, X. *et al.* Substrate engineering of plasmonic nanocavity antenna modes. *Optics Express* **31**, 2345–2358 (2023).

8. Lalanne, P. *et al.* Quasinormal mode solvers for resonators with dispersive materials. *JOSA A* **36**, 686–704 (2019).

9. Yang, L. *et al.* Strong light–matter interactions between gap plasmons and two-dimensional excitons under ambient conditions in a deterministic way. *Nano Letters* **22**, 2177–2186 (2022).

10. Fradkin, I. M., Dyakov, S. A. & Gippius, N. A. Fourier modal method for the description of nanoparticle lattices in the dipole approximation. *Physical Review B* **99**, 075310 (2019).
